# Museum specimens reveal the genomic consequences of long-term population decline in an insect pollinator

**DOI:** 10.1093/molbev/msag223

**Published:** 2026-09-17

**Authors:** Victoria E Mullin, Hana N Merchant, Andres N Arce, Will Nash, David G Notton, Ashleigh Whiffin, Vladimir Blagoderov, Gavin R Broad, James Hogan, Tony Hunter, Simon Jackson, Steve Judd, Jeff Ollerton, Selina Brace, Richard J Gill, Ian Barnes

**Affiliations:** School of Natural Sciences, Trinity College Dublin, Dublin 2 D02 PN40, Ireland; Science Department, Natural History Museum, London SW7 5BD, UK; Science Department, Natural History Museum, London SW7 5BD, UK; Department of Biology, Edge Hill University, Ormskirk L39 4QP, UK; Georgina Mace Centre for the Living Planet, Department of Life Sciences, Imperial College London, Ascot SL5 7PY, UK; School of Biological Sciences, University of East Anglia, Norwich, Norfolk NR4 7TJ, UK; National Museums Scotland, Edinburgh EH1 1JF, UK; National Museums Scotland, Edinburgh EH1 1JF, UK; National Museums Scotland, Edinburgh EH1 1JF, UK; Science Department, Natural History Museum, London SW7 5BD, UK; Oxford University Museum of Natural History, Oxford OX1 3PW, UK; World Museum (National Museums Liverpool), Liverpool L3 8EN, UK; Ipswich Museums (Colchester and Ipswich Museums), Ipswich IP1 3QH, UK; World Museum (National Museums Liverpool), Liverpool L3 8EN, UK; Germplasm Bank of Wild Species and Key Laboratory for Plant Diversity and Biogeography of East Asia, Kunming Institute of Botany, Chinese Academy of Sciences, Kunming 650201, China; School of Life and Environmental Sciences, University of Northampton, Northampton NN1 5PH, UK; Science Department, Natural History Museum, London SW7 5BD, UK; Georgina Mace Centre for the Living Planet, Department of Life Sciences, Imperial College London, Ascot SL5 7PY, UK; Science Department, Natural History Museum, London SW7 5BD, UK

**Keywords:** *Bombus muscorum*, bumblebee, environmental change, heterozygosity, homozygosity, ancient DNA, museum, conservation

## Abstract

Global insect pollinator populations are under threat, with reported declines attributed to increasing habitat loss, pesticide use, and disease. Tracking how genetic diversity has changed over time could reveal the rate and extent of these declines, and the adaptive capacity of affected species—providing an important complement to habitat-based conservation efforts. However, few studies have been able to reconstruct suitable historical baselines to link genomic changes with population change. Here, we use whole genome data from 101 museum specimens of the declining moss carder bumblebee (*Bombus muscorum*) collected across Britain and Ireland between 1894 and 2019 to reveal a dramatic drop in genetic diversity over the last century. We find a substantial (∼24.6%) reduction in genome-wide heterozygosity across Britain during this period. In England and Wales, where habitat fragmentation is most pronounced, we observe a 2.86-fold increase in runs of homozygosity, commensurate with population fragmentation and isolation. Our results reveal the extent to which human-induced environmental change can lead to severe decadal-scale genomic erosion in a functionally important insect. Identified using DNA from historic museum collections, our approach has widespread applicability for insect conservation and understanding the evolutionary consequences of environmental change.

## Introduction

High rates of environmental change over the last century have placed biodiversity under unprecedented pressure (Johnson et al. 2017). For many insect species, factors such as widespread land use change have led to populations becoming fragmented, isolated, and disconnected (Perrin et al. 2025). Recent advances in occupancy modelling and increased availability of open-source survey data support this, showing that the probability of observing important insect groups, like pollinators, has reduced significantly (e.g. Ollerton et al. 2014; Powney et al. 2019; Newbold et al. 2025). Additional factors such as pesticide exposure, climate change, and emergent diseases have also been blamed, with long-term sample trapping supporting this (Woodcock et al. 2016; Hallmann et al. 2017; Fox et al. 2023). However, whilst we have improved our capability to model historic insect occurrences in space and time, our understanding of how such losses are linked to changes in genomic diversity is comparatively poor (Liu et al. 2024). Consequently, we remain relatively ignorant of the related geographical and temporal trends in genetic diversity.

Understanding changes in genome-wide diversity of insect species is important for conservation (Webster et al. 2023). When contemporary populations of a declining species have managed to maintain relatively high genetic diversity, this can indicate they are healthy, resilient, and connected via gene flow (Beaurepaire et al. 2024). In contrast, substantial loss of diversity can signal reduced population viability and provide insights on which factors are driving decline, particularly when the timing of these losses can be established (Jensen et al. 2022). To investigate how population diversity has changed, however, requires historical baseline data with which to compare modern populations (Drake and Griffen 2010; Arce et al. 2023; Sanders et al. 2023). A possible source of such baseline data is through the application of genome sequencing techniques to specimens from museum collections (Mullin et al. 2023). These collections offer an extensive yet underutilised (Kharouba et al. 2018) resource through which we can assess how diversity at the genomic level has changed over time (Wandeler et al. 2007; Díez-Del-Molino et al. 2018). Analyses of DNA from specimens archived over the last century have previously been used to reconstruct historical baseline diversity and to detect inbreeding and infer shifts in population structure (Jensen et al. 2022). However, the development of such approaches has been largely confined to vertebrates (Leonardi et al. 2017), with limited applications to insects (Shpak et al. 2023; Whitla et al. 2024), despite the much larger volume of insect collections in museum storage.

Bumblebees (*Bombus* spp.—Hymenoptera: Apidae) are an important system for testing how genomic diversity is changing; they are critical wild insect pollinators, have declined in many parts of the world, and reference genomes are becoming increasingly available (Woodard et al. 2015). In this study, we focus on the vulnerable (IUCN 2023) moss carder bumblebee, *Bombus muscorum*. *B. muscorum* has a wide but patchy distribution across Eurasia (*Bombus muscorum* (GBIF Secretariat 2023), and although formerly widespread across the British Isles, it is now increasingly confined to mainland Scotland and the Scottish Isles, with some remnant populations around the coast of England and Wales (Darvill et al. 2006). It typically nests in open, flower-rich grasslands, such as meadows and heathland, with a preference for damper habitats, such as marshes and coastal grasslands (Goulson et al. 2006). The species forages on a wide range of different plants for both nectar and pollen, and particularly legumes and other long-corolla flowers (Balfour et al. 2022). *B. muscorum* forms small colonies and has relatively small home ranges and limited migration distances (Darvill et al. 2010). These factors may make it vulnerable to habitat loss, through wetland drainage and agricultural expansion, and to pesticide exposure. Previous work using microsatellites found that current *B. muscorum* populations possess low genetic diversity and exhibit signs of inbreeding (Darvill et al. 2006, 2010). However, the extent and rate of change in genomic diversity remain unknown. It is also unclear whether *B. muscorum* historically maintained higher levels of diversity or whether spatial patterns in genomic diversity have changed over time.

Taking advantage of the recent publication of a high-quality *B. muscorum* genome (Broad et al. 2024), we here apply advances in ancient DNA techniques to leverage historic specimen DNA from bees collected between 1894 and 2019. The main objective of this study is to shed light on the evolutionary consequences of environmental change in a specialist pollinator species. Whilst focusing on a single bumblebee species, our study develops an approach that can, in principle, be applied to any insect collection for multiple purposes, including conservation genetics. We set out to (i) infer broad patterns of population structure within Britain, and whether these have changed over time; (ii) determine the recent and historic levels of genome diversity within the British population; and (iii) establish information about DNA preservation and the accuracy of species identification in museum collections.

## Methods

### Sampling approach

**Figure 1:**
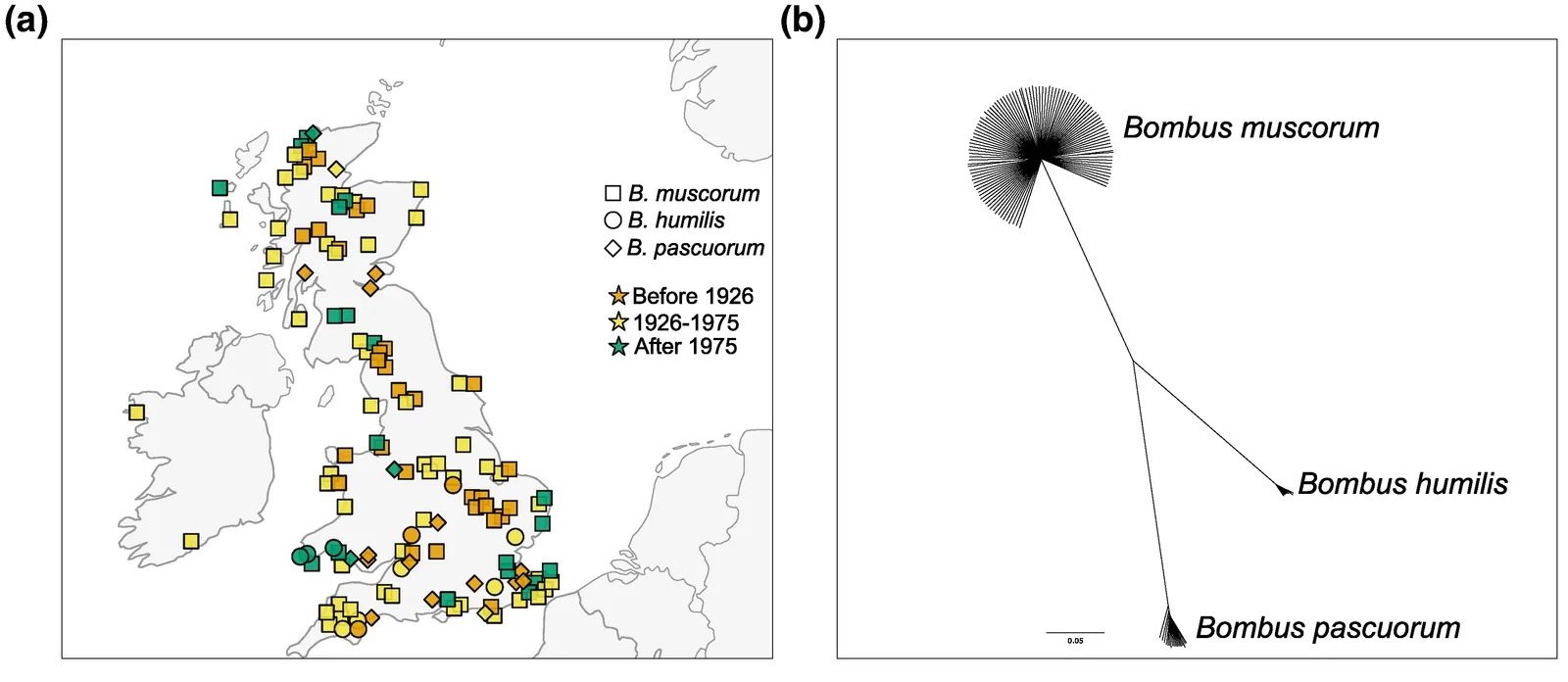
a) Distribution of specimens sampled across Britain and Ireland, grouped into three periodic categories based on the year collected (denoted by colour). Genetically determined species identification is denoted by shape. Points are jittered. b) A whole genome neighbour-joining tree, based on an identity-by-state matrix which demonstrates three distinct species groupings of the samples.

Our sampling was designed to cover the historical range of *B*. *muscorum* in Britain, along with additional samples from the neighbouring population in Ireland, and from the Outer Hebrides where the species is more common (Fig. 1a). Due to the conservation status of *B. muscorum*, sampling was limited to material in historical collections, with the exception of a single specimen collected (postmortem) from South Uist, Outer Hebrides. We used an individual leg per specimen from 130 pinned diploid (worker and queen) individuals, identified as *B. muscorum*, collected between 1890 and 2019, and housed in the collections of five British institutions: Natural History Museum (NHM London), Oxford University Museum of Natural History (OUMNH), Tullie–Museum and Art Gallery (Carlisle), World Museum (Liverpool), and National Museums Scotland (Edinburgh). Of these 130, four individuals (Table S1) were sampled from the Insect Pollinator Initiative (collected between 2011 and 2012) stored at the NHM Molecular Collections Facility. These samples were pinned and air dried, then subsequently moved to the −80 °C storage facility. All specimens were treated as degraded DNA specimens with all pre-PCR laboratory work taking place in the ancient DNA laboratory in the NHM London.

### DNA extraction and library preparation

A modified version of the extraction protocol (Dabney et al. 2013) previously described (Mullin et al. 2023) was performed. In brief, for the lysis stage, 180 μl Qiagen ATL Buffer for tissue lysis and 20 μl Proteinase K were added to each leg and heated at 56 °C for 24 h. DNA purification followed (Dabney et al. 2013), replacing the Zymo-Spin V column binding apparatus with the extender assembly from the High Pure Viral Nucleic Acid Large Volume Kit (Roche). Post purification, to remove postmortem damage, DNA extracts were treated with USER enzyme: 20 μl or 30 μl of extract with 5 μl of USER enzyme for 3 h at 37 °C. Double-stranded libraries were built following (Meyer and Kircher 2010) using AmpliTaq Gold polymerase for the PCR amplification. PCR cycles varied from 13 to 20. Sequencing was performed on an Illumina NextSeq 500 (75 bp PE) and an Illumina NovaSeq 600 (150 bp PE). For the best chance of identifying changes to diversity over the course of the 20th century, we increased sequencing effort for a subset of individuals collected prior to 1925 and after 1975.

### Genome alignment

Sequencing reads were trimmed for adapter sequences using AdapterRemoval v2.3.2 (Schubert et al. 2016), collapse overlapping read pairs with a minimum 11 bp overlap, filter for a minimum read length of 30 bp and trim Ns and low quality bases. Trimmed collapsed reads were aligned using bwa aln (0.7.17-r1198 (Li and Durbin 2009) (-l 1024 -n 0.01 -o 2 -q 15) to the *B. muscorum* reference genome iyBomMusc1.1 (Broad et al. 2024). BAM files were sorted with SAMtools v1.12 (Li et al. 2009), duplicates removed and BAM files merged with Picard (v2.18.7) and mapping quality filtering (q 30) performed with SAMtools (v1.12). Depth of coverage was calculated using Qualimap v.2.1.3 (Okonechnikov et al. 2016) and read length and deamination patterns accessed using mapDamage (Jónsson et al. 2013).

### Genetic verification of samples

Genetic verification via mitochondrial DNA was achieved by applying MITObim (Hahn et al. 2013) to reconstruct the Cytochrome Oxidase I gene (COI), which is the standard for barcode identification of insects (Hebert et al. 2003). A *B. muscorum* COI sequence (GenBank Accession Number: AY181133) was used as the seed, with the parameters ‘—trimreads’, ‘—split’, ‘—clean’, and ‘—quick’. Following this, a Basic Local Alignment Search Tool (BLAST) (Altschul et al. 1990) search was carried out on the mitochondrial assemblies for each sample to obtain the correct species identification using species percentage identity.

For nuclear DNA-based identification, allele sharing between genomes was measured using pairwise identify-by-state (IBS) matrix and computed for 130 samples (Table S1) across the 17 chromosomal pseudomolecules with ANGSD v.0.936 (Korneliussen et al. 2014) -doIBS 1 using the following options; -minMapQ 30 -minQ 25 -GL 1 -doMajorMinor 1 -doMaf 1 -SNP_pval 1e-6 -doCov 1 -doCounts 1 -makeMatrix 1 -minFreq 0.05 -rmTrans 1 -minInd 98 (¾ of samples) -trim 5 -skipTriallelic 1. This retained 560,056 sites.

Individuals assigned to other species were removed from further analyses as were three individuals (VEM 169, 299, 318) belonging to pairs (VEM160—Cromford 1967 and VEM169 Gainsborough 1965, VEM120-Newlands 1974 and VEM299—Newlands 1976, VEM306—Tarn Lodge 1918 and VEM318—Tarn Lodge 1918) identified by IBS as having higher levels of allele sharing suggesting that they were closely related (Figs. 1b, Fig. S1, Table S1), the individual with the lowest coverage in each pair was chosen for removal. Two further individuals (VEM 296 and 305) with negligible levels of heterozygosity were interpreted as males and also removed from all further analyses.

### Population structure of *B. muscorum*

**Figure 2:**
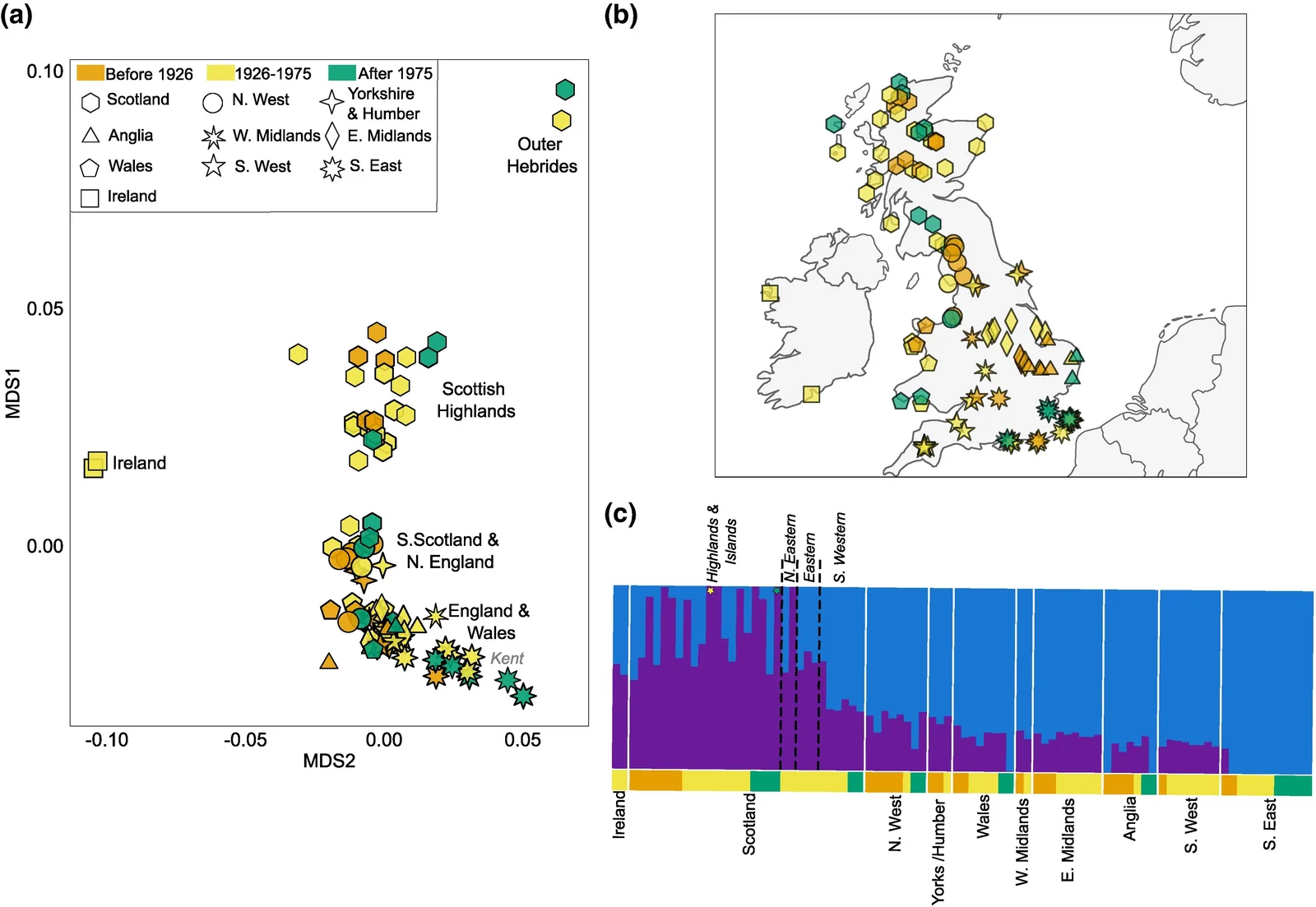
Population structure of *Bombus muscorum* across Britain and Ireland. a) MDS plot constructed from a pairwise identity-by-state matrix of the final 101 *B. muscorum* individuals shows distinct geographical groupings and little temporal structure. b) Distribution of the genetically identified *B. muscorum,* jitter applied. c) Model-based clustering using NGSadmix (K = 2) arranged by the geographic region and ordered temporally within each region. The two outer Hebridian samples are denoted by *.

To compare patterns of allele sharing among the 90 genetically determined *B. muscorum* genomes with an average genome coverage equal to or greater than 0.4× (Table S1), a pairwise identity-by-state (IBS) matrix was computed across the 17 chromosomal pseudomolecules with ANGSD (v.0.936) -doIBS 1 using the following options; -minMapQ 30 -minQ 25 -GL 1 -doMajorMinor 1 -doMaf 1 -doIBS 1 -SNP_pval 1e-6 -doCov 1 -doCounts 1 -makeMatrix 1 -minFreq 0.05 -P 15 -rmTrans 1 -minInd 45 (equivalent to ½ of samples) -trim 5 -skipTriallelic 1 -C 50. This retained 635,486 sites. A classical MDS of the resulting data matrix (Fig. 2a) was computed in R and plotted with ggplot2 (Wickham 2016). The 12 NUTS 1 Geographic Regions of the UK were used for plotting shape purposes.

NGSadmix was used to estimate admixture proportions of low-coverage data (Skotte et al. 2013). Genotype likelihoods were called for the 90 *B. muscorum* genomes with an average genome coverage equal to or greater than 0.4 × (Table S1) across the 17 chromosomal pseudomolecules with ANGSD (v.0.936) using the following options; -minMapQ 30 -minQ 25 -remove_bads 1 -uniqueOnly 1 -trim 5 -rmTrans 1 -doMajorMinor 1 -doMaf 1 -snp_pval 1e-6 -skipTriallelic 1 -minInd 68 (equivalent to ⅔ of samples) -GL 1 -doGlf 2 -doCounts 1 -dumpCounts 1 -setMaxDepth 1000 -C 50 -minmaf 0.05, retaining 421,373 sites. NGSadmix (V.32) was run using a range of K values (1 to 12) and repeated 50 times. CLUMPAK v1.1 (Kopelman et al. 2015) was utilised for both the calculation of the best K (Evanno et al. 2005) and to identify the most frequent clustering solution of the 50 repeats.

### Estimating genetic diversity

Chromosome-level differences in sequencing coverage were assessed using a Kruskal–Wallis rank-sum test, and significant differences in pairwise comparisons were tested using Dunn's tests with Benjamini–Hochberg correction (Fig. S2). Coverage differed significantly among chromosomes (Kruskal–Wallis: H = 2,113.68, df = 16, *P* < 0.001), and posthoc tests showed that chromosomes 4 and 17 had significantly lower coverage than all other chromosomes (adjusted *P* < 0.001), except that chromosome 17 did not differ significantly from chromosomes 6 and 13 (adjusted *P* > 0.05). Consequently, chromosomes 4 and 17 were excluded from analyses of genome-wide heterozygosity (Hz) and runs of homozygosity (ROH).

Heterozygosity was estimated for 32 samples with a minimum average coverage of 3 × (Table S1). Samples of greater average coverage were downsampled to 3 × coverage using the command DownsampleSam in Picard (version 3.2). Hz was calculated in two steps using ANGSD (version 0.936), first site allele frequency likelihoods were calculated (trim 5 -dosaf 1 -GL 1 -minMapQ 30 -minQ 25 -noTrans 1 -remove_bads 1 -uniqueOnly 1 -setMinDepthInd 2 -setMaxDepthInd 8 -doCounts 1 -C 50) followed by estimating the folded site frequency spectrum (realSFS -fold 1). The global heterozygosity was calculated using the fraction of sites that are heterozygous relative to the total number of nonmissing sites in the genome. Using R (version 4.2.0) a linear regression, implemented as a generalised linear model (GLM) with a Gaussian error distribution and an identity link function, was performed to statistically determine the effect of collection date on Hz. To identify potential outlier effects Cook's Distance was conducted, three samples located at the beginning and the end of the timeseries were removed as they exceeded the 4/n threshold (VEM260, VEM319, FM035), and the linear regression rerun.

To estimate ROH, diploid genotypes were called for the 22 samples with a minimum average genome coverage of 6 × (Table S1). Genotypes across the 17 chromosomal pseudomolecules using bcftools (v.1.13) mpileup (-C 50 -q 30 -Q 25 -a FORMAT/AD, FORMAT/SP, FORMAT/ADF,FORMAT/ADR, FORMAT/DP,INFO/AD, INFO/ADF, INFO/ADR) and call (-m -f GQ,GP -v). Calls were filtered using bcftools for biallelic SNPs, removing SNPs within 5 bp of another variant, requiring a depth per variant greater than 50 and less than the sum of the genome average × 2.5, and a minimum quality of 30. Individual genotypes were set to missing if coverage was less than 6×, genotype quality less than 20 and heterozygous positions were subject to an allelic balance window of 25% to 75%. Only transversions with less than 20% of genotypes missing were kept resulting in 631,008 SNPs. In PLINK 1.9 (Chang et al. 2015) the removal of chromosomes 4 and 17, a minor allele count of 2 and a maximum of 5% missingness left 157,015 SNPs.

To identify the regions of the genome that had ROH greater than 50 kb we used PLINK 1.9; --homozyg --homozyg-density 50 --homozyg-gap 200 --homozyg-kb 50 --homozyg-snp 50 --homozyg-window-het 1 --homozyg-window-missing 5 --homozyg-window-snp 50 --homozyg-window-threshold 0.05. In R (version 4.2.0) a linear regression was performed to statistically determine the effect of collection date on the Total Length of ROH and Average length of ROH, Cook's distance was applied to identify outliers. To further evaluate the effect of collection date on the Total length of ROH, a nonparametric Mann–Whitney U test was conducted to test whether the pre-1926 Scottish (n = 2) and English/Welsh (n = 6) individuals could be combined. No significant difference was detected between the two groups (U = 4, *P* = 0.64). Given the absence of a statistically significant difference and the limited sample size of Group 1, the two groups were considered comparable and were pooled. A Kruskal–Wallis test was applied to identify if there is a statistical difference in the Total Length of ROH between these three groupings: pre1926 English/Welsh/Scottish, post1975 English/Welsh, and post1975 Scotland. To determine which pairs of groups differed, a Dunn's posthoc test with Benjamini–Hochberg correct method was conducted.

As a confirmation of the ROH results, visualisation of regions of low diversity was achieved via sliding theta in ANGSD (version 0.936). Site frequency spectrum, previously described, were utilised to calculate thetas for each site (realSFS saf2theta) followed by a sliding window approach (thetaStat windows 50 kb, step 10 kb). Pairwise theta windows values were normalised and visualised in R (version 4.2.0), results are shown for five samples.

#### Estimating genetic load

To estimate changes in the number of deleterious mutations (genetic load) among historical cohorts, we used snpEff (Cingolani et al. 2012) to annotate genetic variants according to their predicted impact on protein function. Variants were classified as *high impact* (likely to severely disrupt protein function, e.g. loss-of-function [LoF] mutations), *moderate impact* (e.g. missense variants), or *low impact* (synonymous substitutions, which may be weakly deleterious given codon-usage bias). For the genetic-load analysis, we used the same set of individuals included in the runs of homozygosity (ROH) analyses (Section Estimating genetic diversity). Diploid genotypes (Section Estimating genetic diversity) were filtered for no missingness across the dataset yielding a total of 612,083 SNPs. Gene annotation (GTF) files were retrieved from Ensembl's Darwin Tree of Life portal (https://projects.ensembl.org/darwin-tree-of-life/). To assess differences in mutation load among cohorts, we fitted linear models for the number of LoF, missense, and synonymous variants per individual. Cohort (*pre-1925*, *post-1975 Scotland*, and *post-1975 England/Wales*) was included as a fixed effect, and average coverage as a covariate to account for differences in sequencing depth. The significance of cohort effects was assessed using ANOVA on the fitted models. All analyses were performed in R v4.5.2.

## Results

### Sequencing of museum specimens

We selected 130 individuals to give a broad spatial and temporal distribution, sampling across the historical range of *B. muscorum* (Fig. 1a). However, nuclear IBS phylogeny and our COI analysis revealed 11 of the specimens to be *B. humilis* and 18 to be *B. pascuorum* (Table S1), leaving us with 101 correctly identified as *B. muscorum* collected between 1894 and 2019. Eleven of the 29 misidentifications were *B. pascuorum* collected by a single individual (A.J. Chitty) between 1890 and 1903 and donated to the OUMNH collection.

For *B. muscorum*, the median coverage was 1.43 × (mean 3.46 ×, range 0.1 ×–15.7 ×; Table S1) with a subset of samples sequenced to higher coverage, including eight samples collected prior to 1925 (median coverage 7.0 ×) and 14 samples collected after 1975 (median coverage 8.6 ×). Reads showed similar levels of postmortem degradation observed previously in museum bumblebees (Mullin et al. 2023) (Fig. S3). Mean read lengths per sample, after alignment, ranged from 48.76 to 89.03, with an average value of 62.74 bp (Fig. S4). There was a significant positive correlation (*F* = 55.28, df = 1 and 99, *P*-value = 3.8 × 10^−11^) between mean read length and age of the sample.

### Spatial and temporal distribution of genetic variation

To explore genetic structure, we performed multidimensional scaling (MDS) on an identity-by-state (IBS) distance matrix across 90 *B. muscorum* samples (Fig. 2; Table S1). Spatial structure was present at both transnational and regional scales, with the MDS plot separating individuals from Ireland and the Outer Hebrides from those in mainland Britain. Within Britain, dimension 1 describes a cline from northern Scotland to southern England (Fig. S5) and dimension 2 varies for individuals found in Kent in south east England. Population structure was largely consistent across the sampling period, with no obvious temporal changes detected. An exception may be the Kent population, where there may be signs of differentiation attributable to genetic drift due to isolation.

To differentiate between the possibility that there were two separate populations in mainland Britain (Scotland vs England and Wales) versus the presence of a single population with a north–south cline, we conducted an admixture analysis using NGSAdmix. CLUMPAK identified K = 2 as the best-supported clustering solution; however, at increasing values of K (3 and 4) ancestry proportions varied gradually along a north–south geographic gradient rather than forming discrete clusters, consistent with isolation-by-distance within a continuously distributed population (Fig. S5). We identify a gradual reduction in northern ancestry components moving southward across Britain, in agreement with the clinal structure observed in the MDS plot (Fig. 2a; Figs. S6 and S7).

### Changes in genetic diversity over time

**Figure 3:**
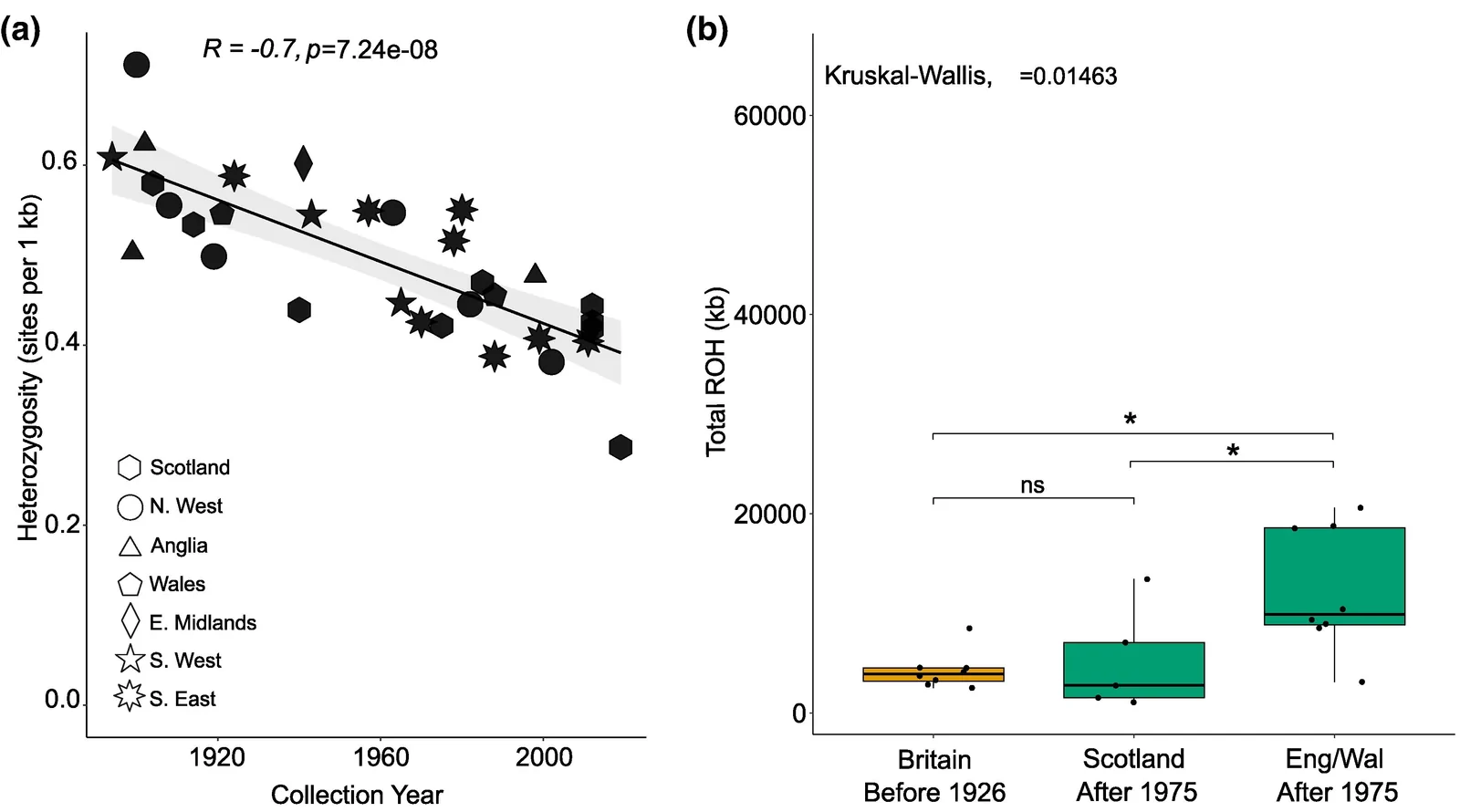
Variation in genome diversity over time for museum samples of *Bombus muscorum*. a) Global heterozygosity (per kb) over time from 32 individuals dated from 1894 to 2019 and caught in regions across Britain. A linear regression line is shown, indicating a significant negative relationship between the collection year and heterozygosity (*P* < 0.001), the shaded band represents the 95% confidence interval. b) Sum of lengths of the genome contained in ROH greater than 50 kb for 21 individuals ranging from 1894 to 2019 across Britain. Both Hz and ROH were calculated excluding chromosomes 4 and 17.

**Figure 4:**
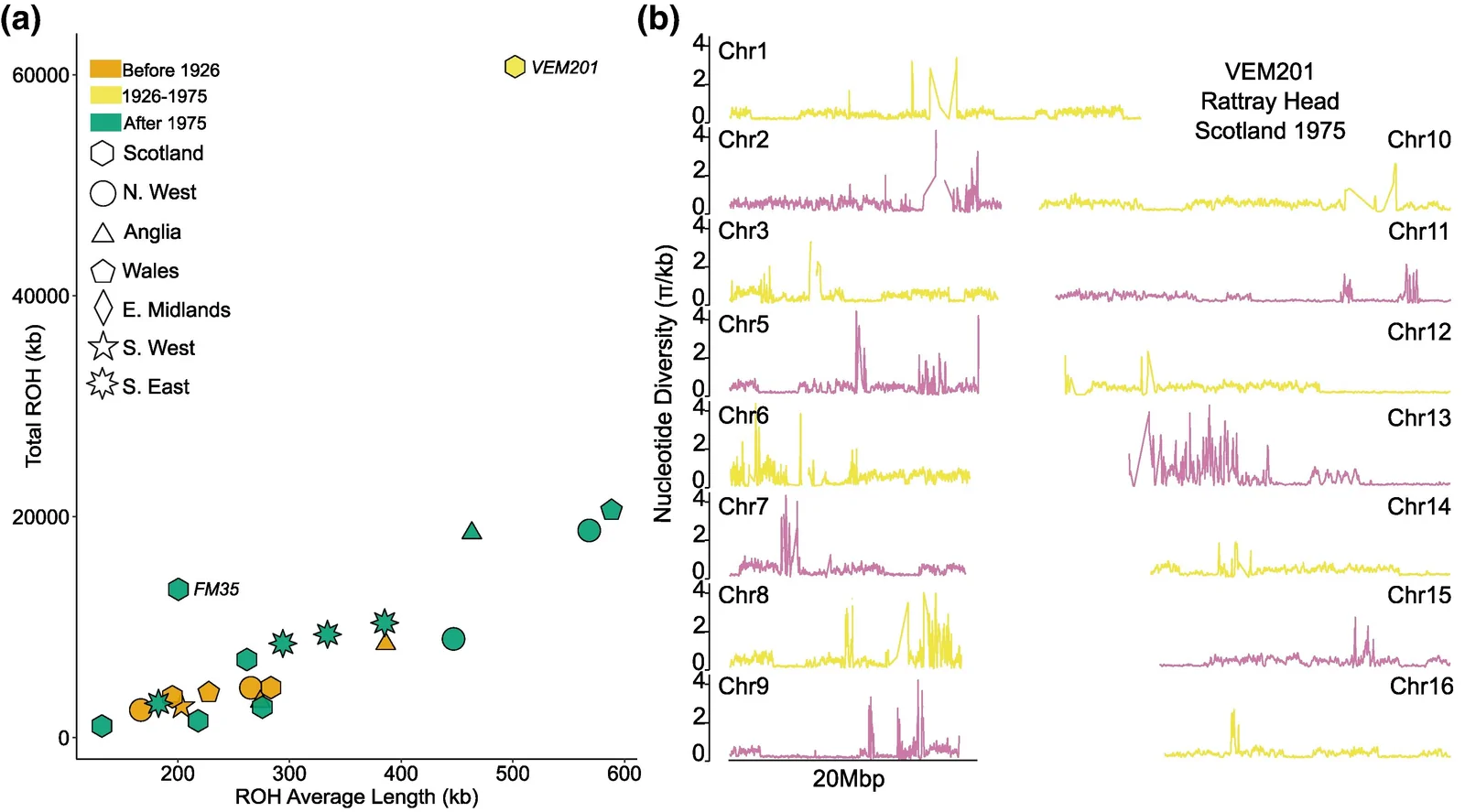
a) Sum of ROH plotted against the average length of ROH for 22 specimens. b) Sliding pairwise theta (50 kb window, 10 kb step) plotted per chromosome of the 1975 Scottish sample VEM201, the outlier from ROH analysis in 4A, visualising regions of low diversity on many of the chromosomes. For comparisons with other samples, see Fig. S9.

Heterozygosity of 32 *B. muscorum* individuals was found to have substantially decreased over the period 1894 to 2019 with a linear regression (*F* = 50.06, df = 1 and 30, *P*-value = 7.24 × 10^−8^) explaining approximately 62.5% of the variance (R^2^ = 0.63, adjusted R^2^ = 0.61 across all regions) (Fig. 3a). The extent of this decline is considerable, decreasing ≈2.82% per decade between 1894 and 2019, and an overall decline of ≈35.3%. This relationship remains significant when three specimens (VEM260, VEM319, and FM035) at the temporal extremes of the time series are excluded as potential outliers (*F* = 46.42 df = 1 and 27, *P*-value = 2.563 × 10^−7^ R^2^ = 0.632, adjusted R^2^ = 0.62, an overall decline of ≈32.2%). This decline in heterozygosity indicates a substantial loss of genome-wide diversity over time; however, heterozygosity alone does not directly measure inbreeding, which we instead assess using runs of homozygosity (ROH) (Fig. 3b, Fig. 4a, Fig. S8).

Time alone does not explain changes in ROH (Fig. S8); When comparing the total ROH of pre-1926 and post-1975 individuals the former group had uniformly low median value of 3902 KB (range 2,502 to 8,496 KB, n = 8), the values have a wider range in the post 1975 cohort (1,055–20,593 KB, n = 13), and a substantially larger median (9,875 KB). However, this pattern—that recent values are more dispersed—seems to derive from differences between Scotland, which includes the Highlands and Outer Hebrides, where the species continues to be more common, compared to constricted populations in England and Wales. We, therefore, grouped our post 1975 samples into two groupings—Scotland vs England/Wales—to look for broad geographical change in total ROH in comparison with the British pre 1926 grouping [Kruskal–Wallis χ^2^(2) = 8.45, *P* = 0.01]. The total sum of ROH post 1975 Scotland remains similar to those across Britain in the early part of the 20th century (*P* = 0.810), whereas we identify a significant (*P* = 0.037) change in values for England and Wales over the same time period. A significant difference is observed between post 1975 Scotland and post 1975 England and Wales (*P* = 0.023).

Furthermore, when comparing total sum of ROH against average length of ROH (Fig. 4a, Fig. S9), earlier Britain-wide and later Scottish individuals demonstrate low total sum ROH and low average length signifying low levels of homozygosity. More recent English and Welsh individuals tend to have accumulated longer averages of ROH and higher total sums, compatible with a reduction in population size due to habitat fragmentation. Two notable Scottish exceptions exist (Fig. 4b, Fig. S9); 1975 Rattary Head (VEM201) has the highest total sum and average length in the dataset and visible regions of reduced diversity suggestive of acute inbreeding. Data for the 2019 Outer Hebridean sample (FM035—South Uist) which has low average ROH length, but an increase in total ROH compared to mainland Scottish samples, suggest long-term population isolation—the island effect. These regional differences in ROH are best interpreted as reflecting spatial variation in demographic history within a geographically structured population, rather than long-standing separation between discrete populations.

Across the 22 historical genomes analysed, snpEff identified 612,083 high-quality biallelic SNPs. The mean numbers of LoF, missense, and synonymous variants per individual were similar among the *pre-1925*, *post-1975 Scotland*, and *post-1975 England and Wales* cohorts (Fig. S10). After accounting for variation in sequencing coverage, cohort had no significant effect on the number of LoF (ANOVA, *P* = 0.881), missense (*P* = 0.364), or synonymous variants (*P* = 0.503).

## Discussion

Given the ongoing uncertainty surrounding changes in global insect diversity (van Klink et al. 2020), our study demonstrates the potential for tracking genome-level change using museum specimens to provide baselines for genome diversity which predate modern farming practices. Here, we find the vulnerable moss carder bumblebee (*B. muscorum*) shows a significant loss of genetic diversity over the last 100 years in Britain. This finding is associated with increasing homozygosity (ROH) values for individuals outside Scotland, consistent with a reduction in effective population size and genetic isolation. Our findings are concerning, especially since we found heterozygosity to continually decline over the last 100 years with no signs of recovery. With such genetic depauperation, populations are potentially at risk to perturbation, disease, and shock events and may lack the ability to adapt to climate change (Whitehorn et al. 2011).

### Population structure

Our analyses support a pattern of isolation-by-distance across Britain, with genomic variation distributed along a north–south cline rather than partitioned into discrete populations. The concordance between the MDS and admixture analyses, together with the absence of strong temporal restructuring, suggests that the British population of *B. muscorum* is better interpreted as a geographically structured continuum. This is in contrast to the buff-tailed bumblebee (*B. terrestris*), a more generalist pollinator which has previously been proposed (Colgan et al. 2022) to have no population structure in Britain. Analysis of runs of homozygosity also suggests a different 20th century population history for Scotland when compared to the rest of Britain. Previous microsatellite studies identified local differentiation within *B. muscorum* populations (Darvill et al. 2006). Our genome-wide analyses are broadly consistent with this observation, but suggest that the underlying structure is more consistent with isolation-by-distance and clinal geographic variation than with sharply discrete population boundaries. Given the low dispersal rates for *B. muscorum*, and the identification of local population differentiation it seems plausible that the current pattern of diversity is being replaced by regional clusters with varying degrees of inbreeding, and by extension, different adaptive potentials and effective population sizes. This has important implications for conservation strategies, particularly the potential for translocation, and raises questions about the level to which such insect population fragmentation is symptomatic of the Anthropocene.

### Genetic diversity change over the century

Our results show that over a period of about 100 years, the British population of *B. muscorum,* including Scotland, has rapidly lost genetic diversity, with a ∼24.6% reduction of genome-wide heterozygosity between the pre-1926 and post-1975 cohorts. For England and Wales, this is accompanied by a ∼186.2% increase in the proportion of the genome contained in runs of homozygosity over 50 kb in England and Wales. Given the species' distribution is contracting across its European range (Edwards and Broad 2005), a loss of genetic diversity was anticipated, but this pace of change is unexpectedly high. It re-emphasises the speed at which rapid agricultural transformation in Britain has impacted pollinator diversity and abundance (Ollerton et al. 2014; Outhwaite et al. 2022).

Previous studies have used museum specimens to explore declines in British pollinators (Wildman et al. 2022; Arce et al. 2023) and their suitability for understanding changes in genetic diversity and how it might inform insect conservation strategies (Mullin et al. 2023). However, the shortage of high-quality reference genomes has limited many of those genetic studies to methods using much smaller numbers of microsatellite loci (Maebe et al. 2015, 2016). Even when reference genomes have been available, the focus has been on evolutionary processes such as extinction (Whitla et al. 2024) or domestication (Espregueira Themudo et al. 2020). Our findings highlight the potential for identifying the rate and extent of change in conservation target species using genome data from museum specimens.

It remains unclear whether genetic diversity loss is confined to some species or represents a more systemic issue being manifested across large parts of the insect tree of life (French et al. 2023). The decline in genetic diversity reported here seems to have occurred progressively over the 20th century and appears to be ongoing, leaving a range of factors, including expansion of agricultural land and long-term reduction in suitable habitats, particularly those with high floral diversity and structural heterogeneity, pesticide usage, and climate change as potential elements in the isolation of these populations (Ollerton et al. 2014).

### Museum insect specimen species identification and sequence read alignment

We found DNA preservation to be similar to previous studies of museum insect specimens (Mullin et al. 2023; Shpak et al. 2023; Whitla et al. 2024), with recovery of genomic information being substantially more consistent than for DNA derived from archaeological specimens. However, we also found that a substantial proportion of specimens (23%) were misidentified, particularly among morphologically similar *Thoracobombus* species. This highlights a broader issue for large-scale analyses using museum catalogue data, where unvalidated identifications may bias inferences of species distributions or ecological trends (Costa et al. 2015).

The *B. muscorum* data exhibited significant variation in alignment of reads to different chromosomes in the reference genome, which we attribute to substantial differences in repetitive DNA between chromosomes. The two most problematic chromosomes in our dataset (removed for analyses where differential genome coverage could impact the output), stand out from the others on the reference genome contact map as having very low (chromosome four) or very high (chromosome 17) contact with the rest of the genome (Broad et al. 2024) which suggests that the chromosomal contact maps could be used as a screening tool in future work. The presence of repetitive elements in *Bombus* is increasingly recognised, with examples including a cross-clade transposable element (Madrigal et al. 2025) and a 10-mer repeat (Crowley et al. 2023), but both the proportion of repetitive DNA, and variation across the genome seem to be larger in *B. muscorum* than in other *Bombus* species (Table S2), which may indicate reduced efficacy of purifying selection associated with reductions in Ne.

### Implications for the conservation status of the moss carder bee

Across its European range, populations of *B. muscorum* are generally isolated, and the species has been lost from parts of its range, leading to its recent IUCN Red List assessment as Vulnerable (IUCN 2023). In the UK, *B. muscorum* has declined considerably since the 19th century (Bees et al. 2025) and is listed as a Species of Principal Importance under Sections 41 and 42 of the Natural Environment and Rural Communities (NERC) Act 2006 for England and Wales. Public bodies must, therefore, have due regard for the impact of their decisions on this species and its habitat, for example, in planning applications. Our results indicate substantial erosion of genetic diversity in *B. muscorum*, particularly in England and Wales, over the past century, consistent with increasing isolation and reduced gene flow. Although our temporal sampling does not allow a direct evaluation of the effectiveness of very recent conservation measures, the continued decline in heterozygosity suggests that existing populations may remain vulnerable to further genetic erosion. This pattern highlights the importance of strengthening conservation efforts aimed at restoring habitat connectivity and supporting viable population sizes. In fragmented populations showing strong regional genetic structure, conservation translocations or assisted gene flow have sometimes been proposed as a means of restoring connectivity and reducing inbreeding. For pollinating insects, including bumblebees, the movement of individuals between populations could theoretically increase genetic diversity in isolated populations, although this would need careful assessment of habitat suitability and potential disease transmission risks (Webster et al. 2023).

## Conclusion

Concerns over biodiversity loss in the Anthropocene have largely focused on taxonomic diversity, with spatiotemporal changes in genomic diversity remaining underexplored—particularly among invertebrates. Yet, detecting shifts in invertebrate population structure and genome-wide diversity can provide critical insight into the timing and drivers of regional or national declines with significant implications for ecosystem functioning. Here, we demonstrate the utility of museum specimens for revealing and quantifying changes in genomic diversity and assessing the genetic health of extant populations. Insect collections offer distinct advantages over those of birds and mammals: they are typically far more extensive, possess smaller genomes that are cheaper to sequence, and often include precise collection dates. These features make them especially valuable for conservation research (Davis et al. 2021) and the study of evolutionary dynamics. We advocate for broader integration of ancient genomics and museomics to track and elucidate the processes underlying genomic erosion across insect communities for different biomes and natural versus modified landscapes.

## Supplementary Material

msag223_Supplementary_Data

## Data Availability

The raw sequencing data is available via the European Nucleotide Archive (ENA) under the accession number PRJEB55244.
