# Supplementary material for: Museum specimens reveal the genomic consequences of long-term population decline in an insect pollinator
Source: Mol Biol Evol. 2026 Sep 17;43(9):msag223. doi: 10.1093/molbev/msag223 (PMC13582021; doi:10.1093/molbev/msag223)
Supplement: Supplementary file 1 [file msag223_supplementary_data.zip › Bmuscorum_SI_MBE_resubmission.pdf]

Supplementary information

A Century of Population Decline in an Insect Pollinator Revealed by Museum Genomes.

Victoria E Mullin, Hana N Merchant, Andres N Arce, Will Nash, David G Notton, Ashleigh Whiffin, Vladimir Blagderov, Gavin R Broad, James Hogan, Tony Hunter, Simon Jackson, Steve Judd, Jeff Ollerton, Selina Brace, Richard J Gill, Ian Barnes

Table Descriptions

Table S1: Sample information for the whole dataset of *Bombus muscorum* museum samples (n= 131). For each sample we provide Museum Name (specimen identifier in museum's database) or project code, Lab Name (NHM lab code), Museum, Museum Species (species identification in the museum catalogue, within *Bombus*), MITObim Genetic Species (if different to museum catalogue identification), Caste, Date of Collection, Location, County, Region, Extra Region Info, Latitude and Longitude, Notes, Coverage, and information on which analyses the samples was used in (MITObim, IBS Tree, MDS, Heterozygosity, Diploid Calls and Sum ROH>50KB).

Table S2: Proportion of repetitive DNA by chromosome for 10 Bombus species, determined from Repeatmodeller outputs (see https://projects.ensembl.org/darwin-tree-of-life/).

Figures

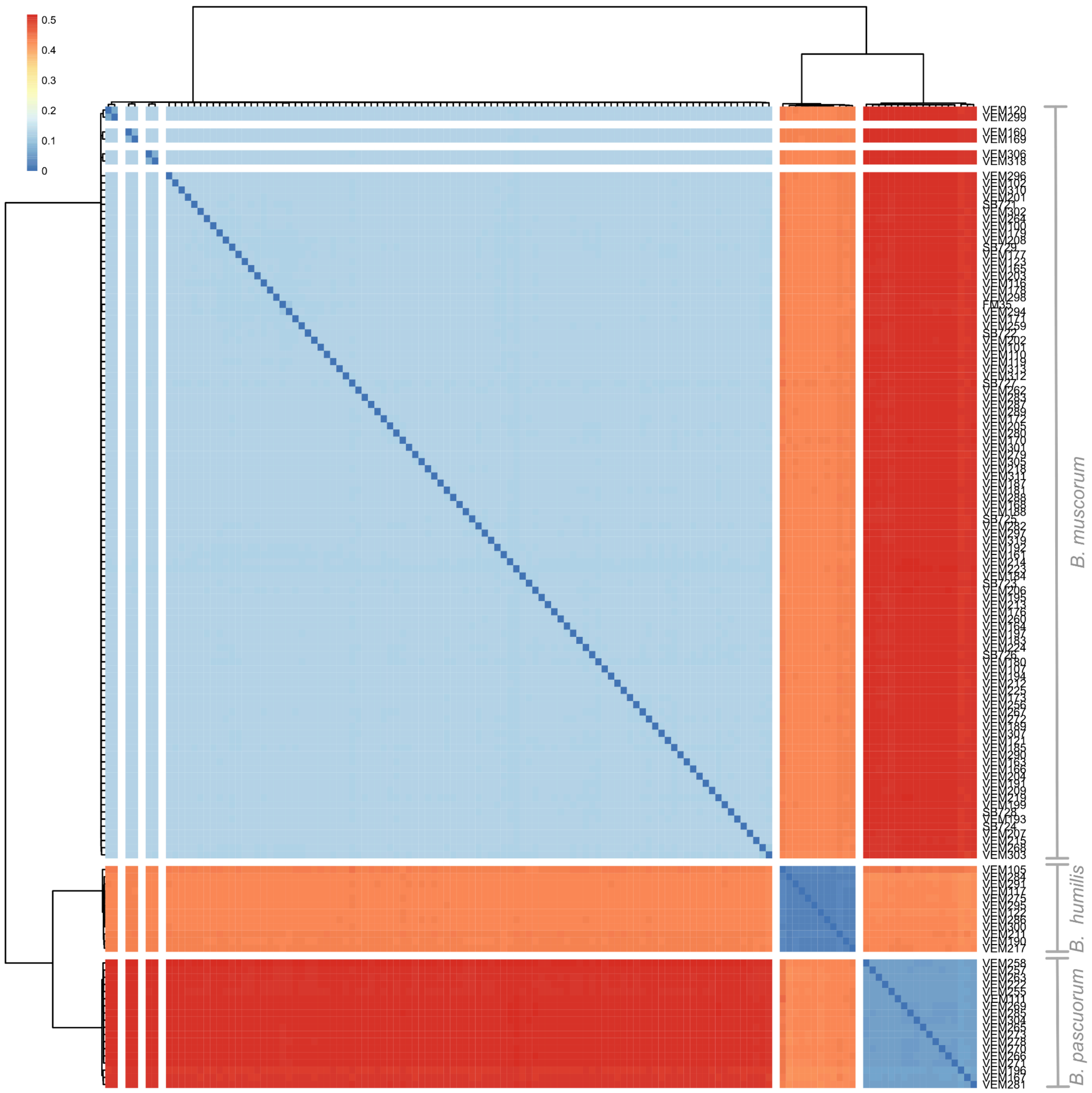

Figure S1: Identity-by-state demonstrates the three species groupings and within *Bombus muscorum* three pairs of samples show higher levels of allele sharing than the rest of *B. muscorum* samples. Within these three pairs samples are close in age and geography (SI Table 1).

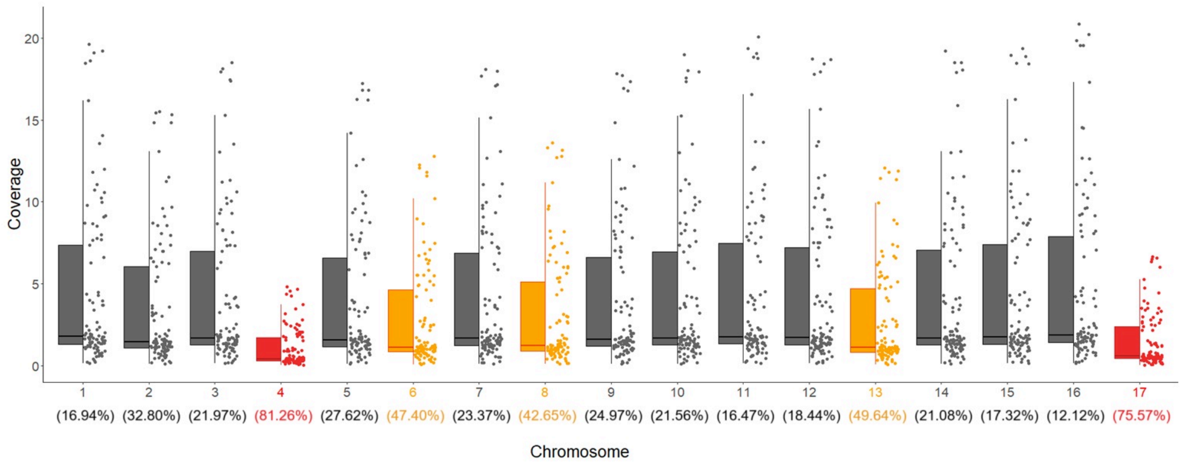

Figure S2: Box and jitter plot showing the chromosome coverage of the 101 *Bombus muscorum* samples used in this study, for all 17 chromosomes. Percentage repeat DNA values are presented in brackets below each chromosome number, based on softmasked repeat data of the *B. muscorum* reference genome from ENSEMBL (https://projects.ensembl.org/darwin-tree-of-life/). Chromosomes 4 (81.26%) and 17 (75.57%) are highlighted in red and are the two chromosomes identified as having significantly lower coverage than other chromosomes ($\chi^2(116) = 2113.68$, $p < 0.001$), and have a high proportion of repetitive DNA. Chromosomes 6 (47.40%), 8 (42.65%) and 13 (49.64%), highlighted in orange, also show reduced coverage and high proportion of repetitive DNA.

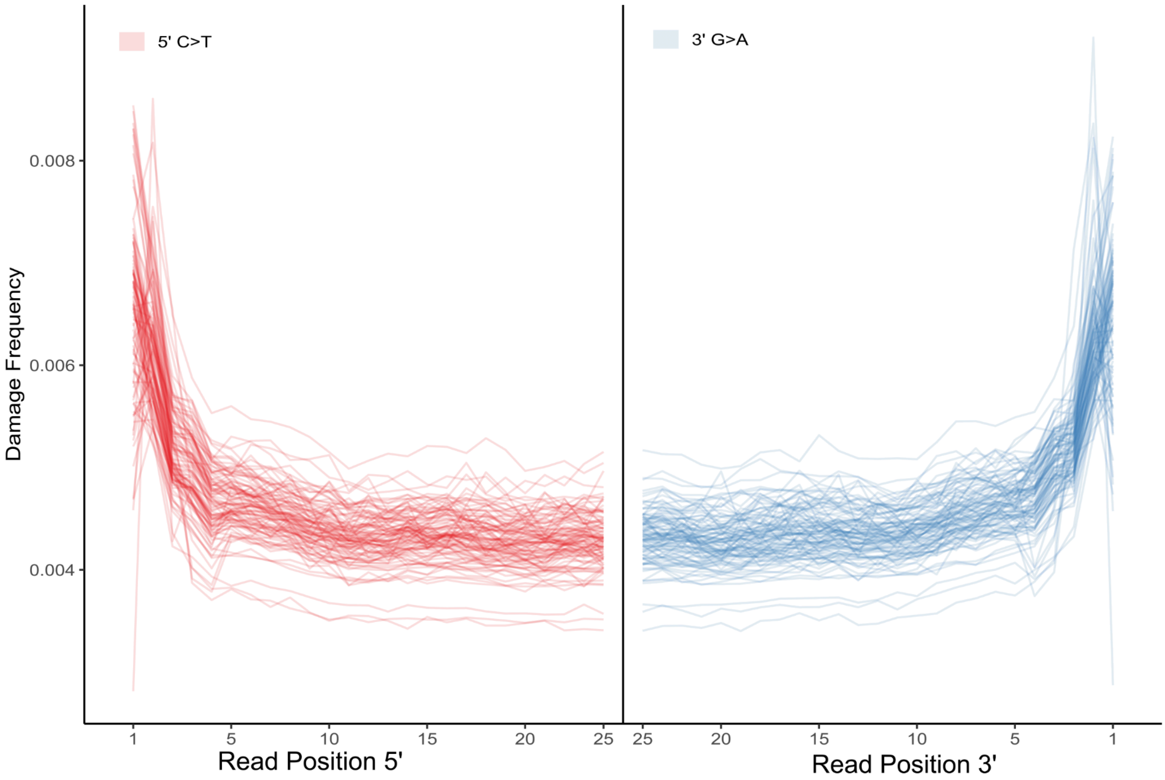

Figure S3: Deamination patterns for each *Bombus muscorum* individual at the 5’ and 3’ prime end of reads. All samples were USER treated.

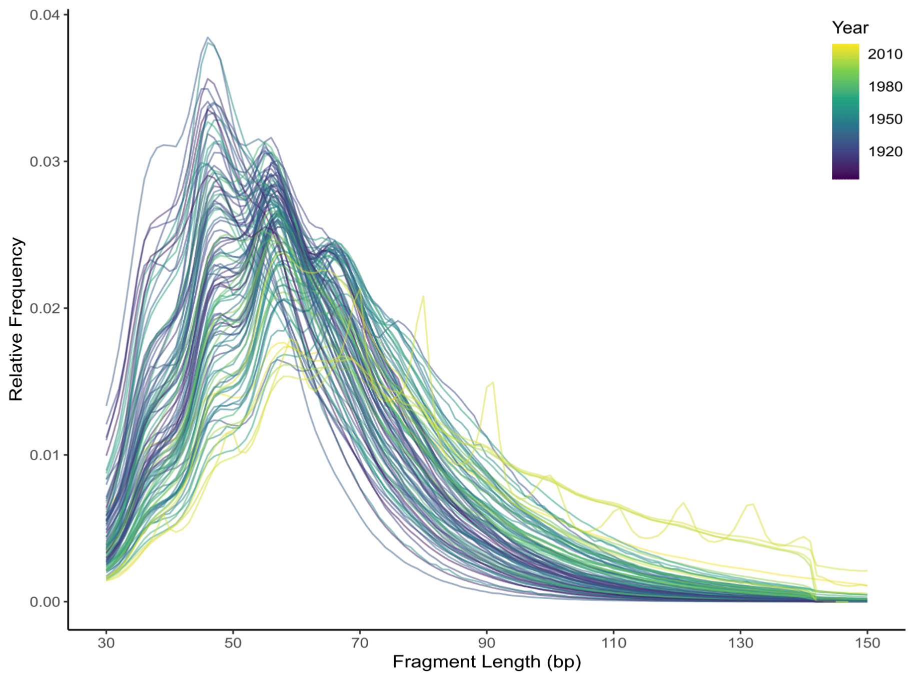

Figure S4: Read length distributions for all *Bombus muscorum* genomes.

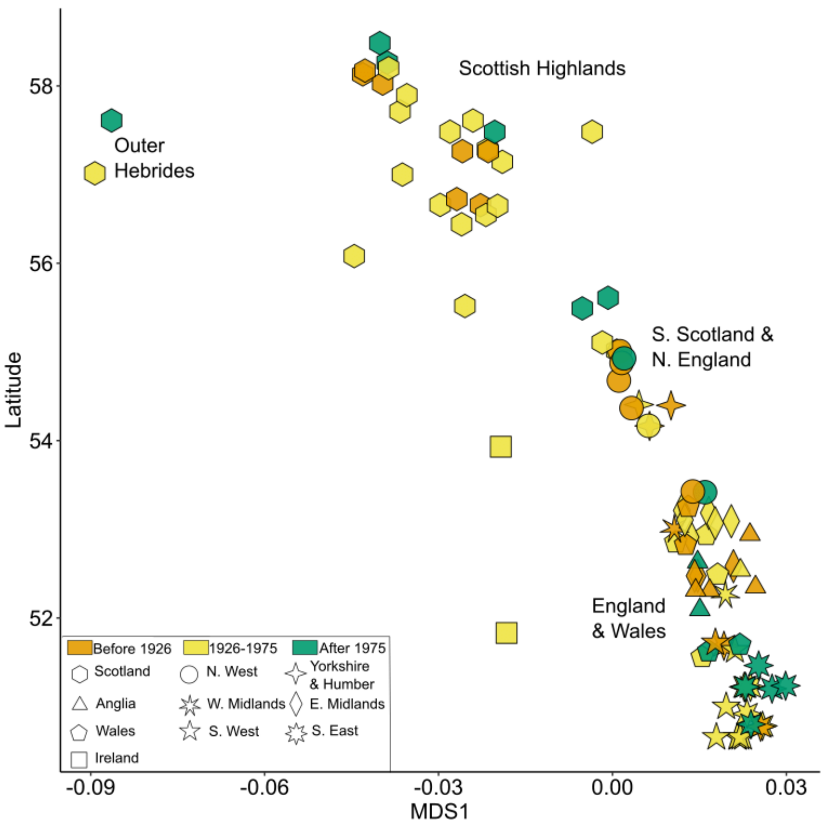

Figure S5: MDS Dimension 1 vs. Latitude. A strong negative correlation calculated with spearman's rank identifies a clear latitudinal cline (ρ = -0.915, p< 2.2 x 10^{-16} , n = 90) .

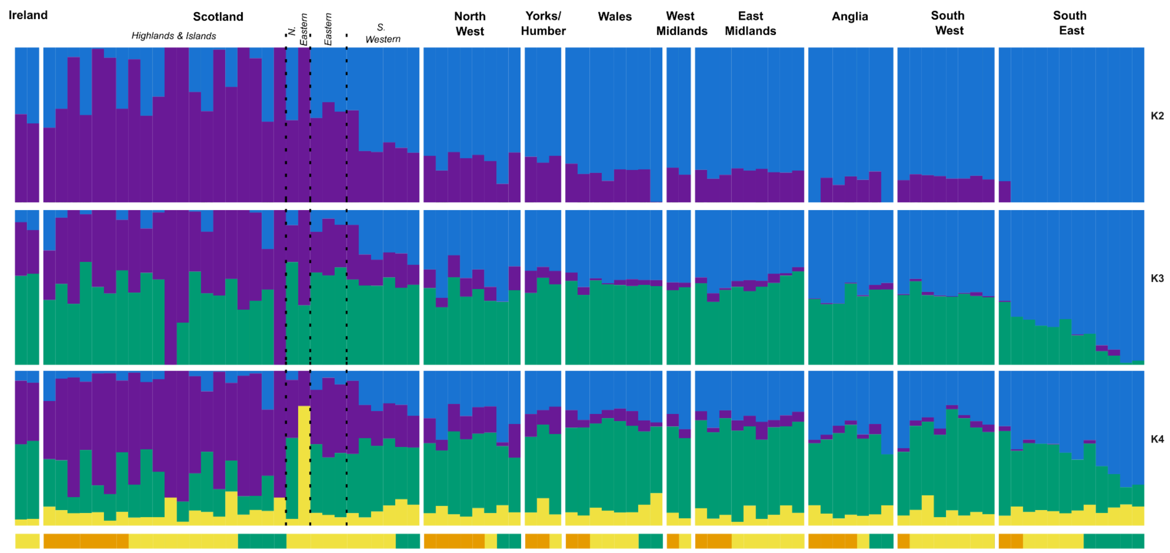

Figure S6: Model-based clustering using NGSadmix (K=2-4) arranged by geographic region and ordered temporally within each region. Temporal groupings as per MDS (Figure 2A, SI Fig. 5) are shown along the bottom. Repeat runs were merged by CLUMPAK and the major cluster - representing the most frequent solution - was plotted. K=2-3 demonstrate a

geography cline in ancestry. K=4 the fourth ancestry is dominated by VEM201- a Scottish sample demonstrating patterns of acute inbreeding.

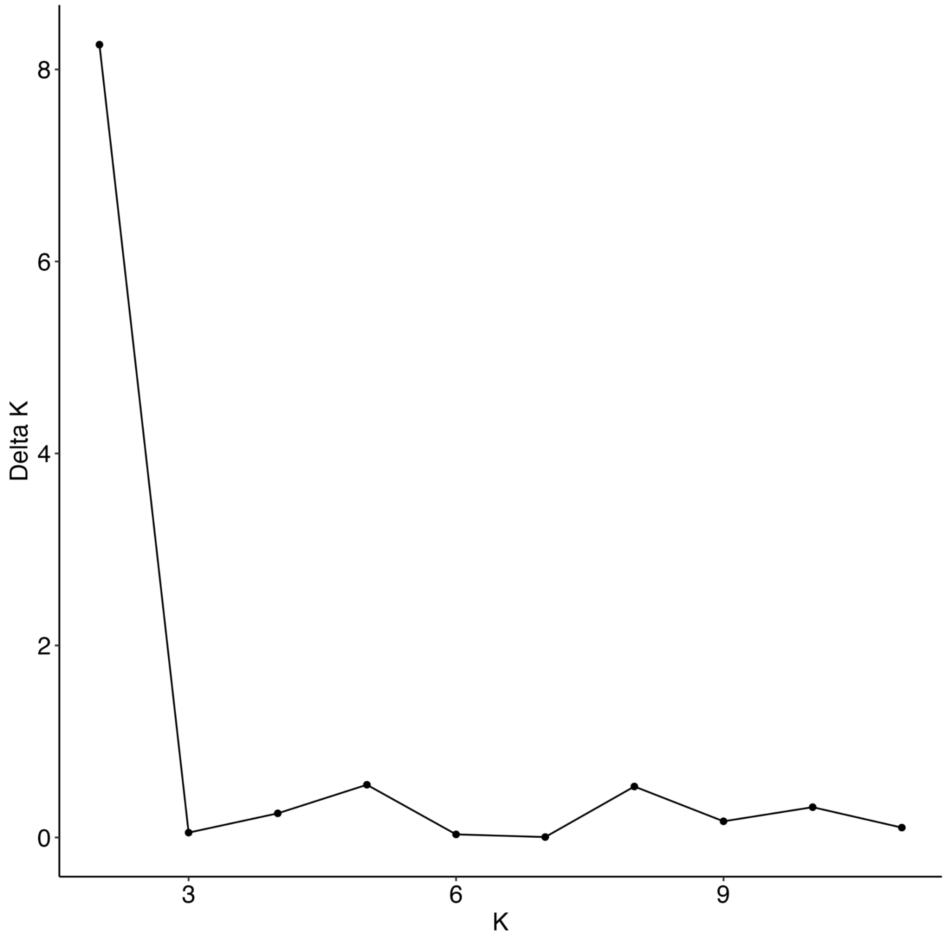

Figure S7: Best K for NGSadmix calculated using Evanno Method in CLUMPAK.

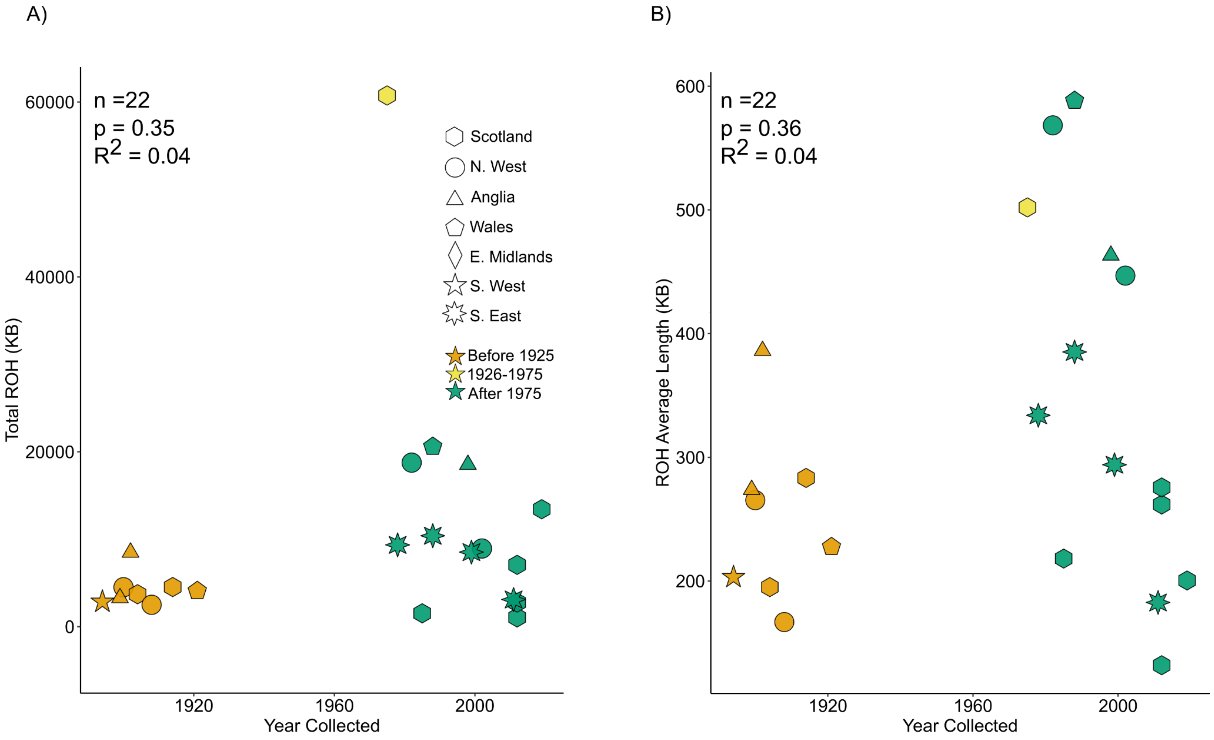

Figure S8: A) Total ROH (KB) plotted against specimen collection year. No significant linear correlation was found between collection year and total ROH length (linear regression, p = 0.35, R^2 = 0.04, n = 22). When the outlier VEM201 (Cook's D = 0.4) is removed, there remains no significant linear correlation (p = 0.11, R^2 = 0.13, n = 21). **B)** Average ROH Length plotted against specimen collection year. No significant linear correlation was detected (linear regression, p = 0.36, R^2 = 0.04, n = 22).

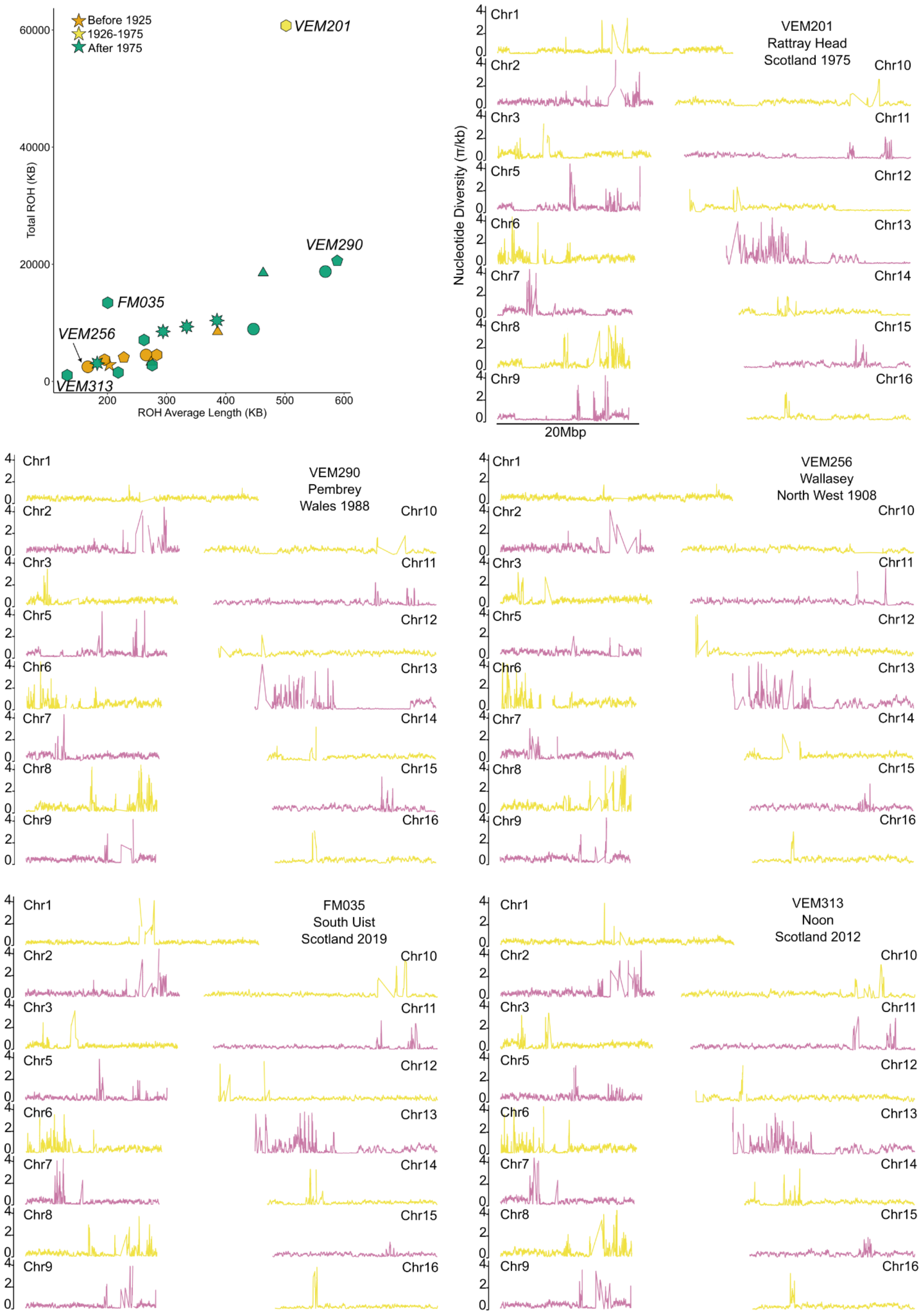

Figure S9: Sliding pairwise theta (50kb window, 10kb step) plotted per chromosome for VEM201, VEM256, FM035, VEM313 and VEM290. Samples were picked to demonstrate the sliding theta results for different values of Total ROH vs Average Length. Notably, regions of low homozygosity can be distinguished in VEM201 and VEM290.

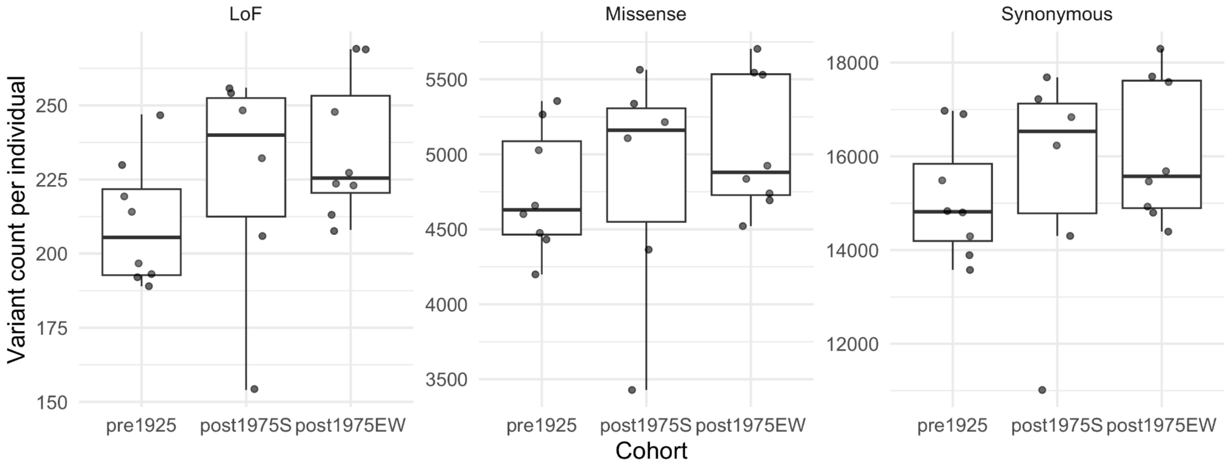

Figure S10: Genetic load estimates among historical *Bombus muscorum* cohorts. Box-and-jitter plots showing the number of (a) loss-of-function (LoF), (b) missense, and (c) synonymous variants per individual in the *pre-1925*, *post-1975 Scotland*, and *post-1975 England and Wales* cohorts. Boxes indicate the interquartile range, centre lines indicate medians, and points represent individual genomes. Variant classes were assigned using snpEff annotation. No significant differences in the numbers of LoF, missense, or synonymous variants were detected among cohorts after accounting for variation in sequencing coverage.
